# 4-(4-Fluoro­phen­yl)-6-(2-fur­yl)pyrimidin-2-amine

**DOI:** 10.1107/S1600536808012294

**Published:** 2008-05-03

**Authors:** Mujahid Hussain Bukhari, Hamid Latif Siddiqui, Muhammad Ashraf Chaudhary, Tanvir Hussain, Masood Parvez

**Affiliations:** a Institute of Chemistry, University of the Punjab, Lahore 54590, Pakistan; b Department of Chemistry, F. C. College University, Lahore 54600, Pakistan; c Department of Chemistry, The University of Calgary, 2500 University Drive NW, Calgary, Alberta, Canada T2N 1N4

## Abstract

Mol­ecules of the title compound, C_14_H_10_FN_3_O, are essentially planar and in the crystal structure they form dimers *via* hydrogen bonds, involving pyrimidinyl N atoms and amino H atoms, about inversion centers. The centroids of the furyl and pyrimidinyl rings are separated by 3.489 (2)Å, indicating π–π stacking inter­actions.

## Related literature

For related literature, see: Colorado, & Brodbelt (1996 ▶); Bojarski *et al.* (1985 ▶); Fun *et al.* (2006 ▶); Gallagher *et al.* (2004 ▶); Hueso *et al.* (2003 ▶); Miranda *et al.* (2006 ▶); Varga *et al.* (2003 ▶).; Miyazaki *et al.* (2005 ▶).
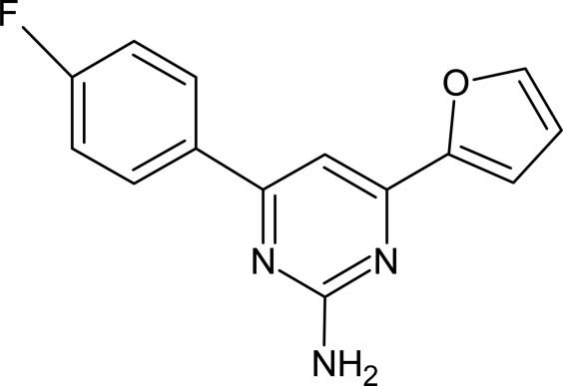


## Experimental

### 

#### Crystal data

C_14_H_10_FN_3_O
*M* *_r_* = 255.25
Monoclinic, 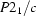

*a* = 11.629 (4) Å
*b* = 5.992 (3) Å
*c* = 16.389 (6) Å
β = 97.69 (2)°
*V* = 1131.7 (8) Å^3^
*Z* = 4
Mo *K*α radiation
μ = 0.11 mm^−1^
*T* = 173 (2) K
0.24 × 0.20 × 0.16 mm

#### Data collection

Nonius KappaCCD diffractometer
Absorption correction: multi-scan (*SORTAV*; Blessing, 1997 ▶) *T*_min_ = 0.974, *T*_max_ = 0.983
4617 measured reflections
2567 independent reflections
1935 reflections with *I* > 2σ(*I*)
*R*_int_ = 0.033

#### Refinement

*R*[*F* ^2^ > 2σ(*F* ^2^)] = 0.044
*wR*(*F* ^2^) = 0.123
*S* = 1.03
2567 reflections
179 parameters
H atoms treated by a mixture of independent and constrained refinement
Δρ_max_ = 0.25 e Å^−3^
Δρ_min_ = −0.21 e Å^−3^

### 

Data collection: *COLLECT* (Hooft, 1998 ▶); cell refinement: *DENZO* (Otwinowski & Minor, 1997 ▶); data reduction: *SCALEPACK* (Otwinowski & Minor, 1997 ▶); program(s) used to solve structure: *SHELXS97* (Sheldrick, 2008 ▶); program(s) used to refine structure: *SHELXL97* (Sheldrick, 2008 ▶); molecular graphics: *ORTEP-3 for Windows* (Farrugia, 1997 ▶); software used to prepare material for publication: *SHELXL97*.

## Supplementary Material

Crystal structure: contains datablocks global, I. DOI: 10.1107/S1600536808012294/lh2621sup1.cif

Structure factors: contains datablocks I. DOI: 10.1107/S1600536808012294/lh2621Isup2.hkl

Additional supplementary materials:  crystallographic information; 3D view; checkCIF report

## Figures and Tables

**Table 1:** Hydrogen-bond geometry (Å, °)

*D*—H⋯*A*	*D*—H	H⋯*A*	*D*⋯*A*	*D*—H⋯*A*
N3—H31⋯N2^i^	0.90 (2)	2.30 (2)	3.190 (2)	168 (2)
C5—H5⋯O1^ii^	0.95	2.58	3.474 (2)	157
C2—H2⋯N1	0.95	2.46	2.789 (2)	100

Symmetry codes: (i) 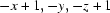; (ii) 
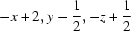
.

**Fig. 1.**
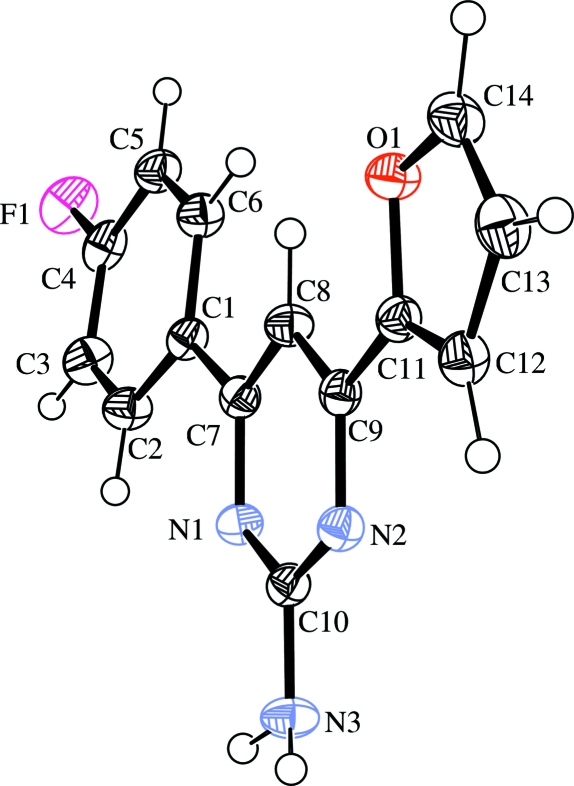
ORTEP-3 (Farrugia, 1997) drawing of (I) with displacement ellipsoids plotted at 50% probability level.

**Fig. 2.**
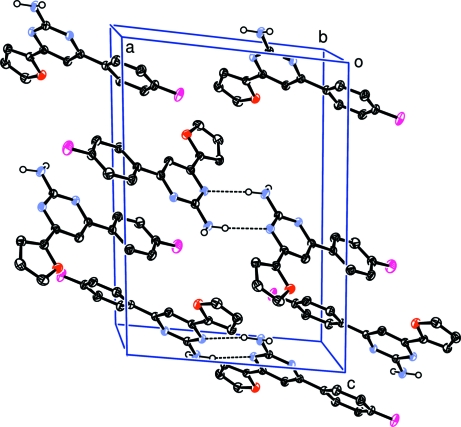
Unit cell packing of (I) showing hydrogen bonds with dashed lines; H-atoms not involved in H-bonds have been omitted.

## supplementary crystallographic information

### Comment

Compounds containing a pyrimidine moiety play a significant role in many biological systems (Hueso *et al.*, 2003). The pyrimidine ring is present in nucleic acids, several vitamins, coenzymes and antibiotics. Pyrimidine based compounds have been reported as anticancer and antiviral agents (Miyazaki *et al.*, 2005). They have been used as hypnotic drugs for the nervous system, *e.g.*, barbiturates act as anaesthetic and sleeping agents and have been in use for the treatment of anxiety, epilepsy and other psychiatric disorders (Colorado & Brodbelt, 1996; Bojarski *et al.*, 1985). We have prepared a series of pyrimidine based compounds from different chalcones following the literature method (Varga *et al.*, 2003). In this paper we report the preparation and structure of the title pyrimidine compound, (I).

The crystal structure of (I) is composed of more or less planar molecules of 4-(4-fluorophenyl)-6-(2-furyl)pyrimidin-2-yl-amine (Fig. 1) wherein the dihedral angle between the mean-planes formed by the furyl and pyrimidinyl rings is 1.91 (12)° and the phenyl ring is oriented at 12.33 (11) and 10.45 (10)° with respect to these rings, respectively. The atoms F1 and N3 are displaced from the mean-planes of the phenyl and pyrimidinyl rings by 0.026 (2) and 0.032 (2) Å, respectively. The bond distances and bond angles in (I) agree well with the corresponding bond distances and angles reported in some compounds closely related to (I)(*e.g.*, Gallagher *et al.*, 2004; Fun *et al.*, 2006; Miranda *et al.*, 2006). The geometry at atom N3 is trigonal pyramidal with sum of the angles about N3 being 348.6°.

It is interesting to note that only one of the amino H-atoms, namely H31 is involved in hydrogen bonding, resulting in dimers about inversion centers (Fig. 2) (details of hydrogen bonding geometry are given in Table 1). In addition, non-classical intermolecular hydrogen bonds, C5–H5···O1, and intramolecular interactions C2–H2···N1 were also observed. The shortest distance between the centroids of furyl and pyrimidinyl rings from adjacent molecules separated by translation along the *b* axis is 3.489 (2) Å indicating π-π stacking interactions.

### Experimental

3-(4-Fluorophenyl)-1-(furan-2-yl)prop-2-en-1-one (2.5 g, 9.08 mmol), guanidine hydrochloride (1.3 g, 1.5 mmol), ethanol (20 ml) and 50% aqueous KOH solution (4 ml) were mixed together and stirred at reflux temperature for 1 hr. Under the same conditions, 30% aqueous H_2_O_2_ (3.1 ml, 27.3 mmol) was added to the above mixture in small portions over a period of I hr. The ethanol was removed under reduced pressure in a rotary evaporator and distilled water (20 ml) was added to the residue. The product was isolated as precipitates, washed repeatedly with pure water and recrystallized from chloroform (yield 58%).

### Refinement

Though all the H atoms could be distinguished in the difference Fourier map the H-atoms bonded to C-atoms were included at geometrically idealized positions and refined in riding-model approximation with the following constraints: C—H distances were set to 0.95 Å and *U*_iso_(H) = 1.2*U*_eq_(C). H-atoms bonded to N3 were taken from the difference map and were allowed to refine with *U*_iso_ = 1.2 times *U*_eq_ of N3. The final difference map was free of any chemically significant features.

### Crystal data

**Table:**

C_14_H_10_FN_3_O	*F*_000_ = 528
*M**_r_* = 255.25	*D*_x_ = 1.498 Mg m^−^^3^
Monoclinic, *P*2_1_/*c*	Melting point = 513–515 K
Hall symbol: -P 2ybc	Mo *K*α radiation λ = 0.71073 Å
*a* = 11.629 (4) Å	Cell parameters from 4617 reflections
*b* = 5.992 (3) Å	θ = 3.6–27.5º
*c* = 16.389 (6) Å	µ = 0.11 mm^−^^1^
β = 97.69 (2)º	*T* = 173 (2) K
*V* = 1131.7 (8) Å^3^	Prism, colorless
*Z* = 4	0.24 × 0.20 × 0.16 mm

### Data collection

**Table:**

Nonius KappaCCD diffractometer	2567 independent reflections
Radiation source: fine-focus sealed tube	1935 reflections with *I* > 2σ(*I*)
Monochromator: graphite	*R*_int_ = 0.033
*T* = 173(2) K	θ_max_ = 27.5º
ω and φ scans	θ_min_ = 3.6º
Absorption correction: multi-scan(SORTAV; Blessing, 1997)	*h* = −14→15
*T*_min_ = 0.974, *T*_max_ = 0.983	*k* = −7→7
4617 measured reflections	*l* = −21→21

### Refinement

**Table:**

Refinement on *F*^2^	Hydrogen site location: inferred from neighbouring sites
Least-squares matrix: full	H atoms treated by a mixture of independent and constrained refinement
*R*[*F*^2^ > 2σ(*F*^2^)] = 0.044	*w* = 1/[σ^2^(*F*_o_^2^) + (0.061*P*)^2^ + 0.4*P*] where *P* = (*F*_o_^2^ + 2*F*_c_^2^)/3
*wR*(*F*^2^) = 0.123	(Δ/σ)_max_ < 0.001
*S* = 1.03	Δρ_max_ = 0.25 e Å^−^^3^
2567 reflections	Δρ_min_ = −0.21 e Å^−^^3^
179 parameters	Extinction correction: SHELXL97 (Sheldrick, 2008), Fc^*^=kFc[1+0.001xFc^2^λ^3^/sin(2θ)]^-1/4^
Primary atom site location: structure-invariant direct methods	Extinction coefficient: 0.025 (6)
Secondary atom site location: difference Fourier map	

### Special details

**Table:**

Geometry. All e.s.d.'s (except the e.s.d. in the dihedral angle between two l.s. planes) are estimated using the full covariance matrix. The cell e.s.d.'s are taken into account individually in the estimation of e.s.d.'s in distances, angles and torsion angles; correlations between e.s.d.'s in cell parameters are only used when they are defined by crystal symmetry. An approximate (isotropic) treatment of cell e.s.d.'s is used for estimating e.s.d.'s involving l.s. planes.
Refinement. Refinement of *F*^2^ against ALL reflections. The weighted *R*-factor *wR* and goodness of fit *S* are based on *F*^2^, conventional *R*-factors *R* are based on *F*, with *F* set to zero for negative *F*^2^. The threshold expression of *F*^2^ > σ(*F*^2^) is used only for calculating *R*-factors(gt) *etc*. and is not relevant to the choice of reflections for refinement. *R*-factors based on *F*^2^ are statistically about twice as large as those based on *F*, and *R*- factors based on ALL data will be even larger.

### Fractional atomic coordinates and isotropic or equivalent isotropic displacement parameters (Å^2^)

**Table:**

	*x*	*y*	*z*	*U*_iso_*/*U*_eq_	
F1	1.25524 (8)	−0.3651 (2)	0.33665 (7)	0.0417 (3)	
N1	0.78951 (10)	−0.1133 (2)	0.47173 (8)	0.0212 (3)	
N2	0.63490 (10)	0.1576 (2)	0.46105 (8)	0.0214 (3)	
N3	0.64102 (12)	−0.1403 (3)	0.55076 (8)	0.0260 (3)	
H31	0.5641 (17)	−0.128 (3)	0.5524 (11)	0.031*	
H32	0.6685 (16)	−0.273 (4)	0.5618 (11)	0.031*	
O1	0.68164 (9)	0.57384 (19)	0.30924 (7)	0.0252 (3)	
C1	0.94793 (12)	−0.0974 (3)	0.39086 (9)	0.0209 (3)	
C2	0.98310 (14)	−0.3102 (3)	0.41767 (10)	0.0269 (4)	
H2	0.9353	−0.3948	0.4488	0.032*	
C3	1.08664 (14)	−0.4012 (3)	0.39983 (10)	0.0302 (4)	
H3	1.1108	−0.5456	0.4191	0.036*	
C4	1.15313 (13)	−0.2769 (3)	0.35368 (10)	0.0282 (4)	
C5	1.12145 (14)	−0.0680 (3)	0.32395 (10)	0.0280 (4)	
H5	1.1690	0.0130	0.2915	0.034*	
C6	1.01768 (13)	0.0212 (3)	0.34272 (10)	0.0256 (4)	
H6	0.9939	0.1649	0.3225	0.031*	
C7	0.83900 (12)	−0.0007 (3)	0.41450 (9)	0.0203 (3)	
C8	0.79028 (13)	0.1937 (3)	0.37932 (9)	0.0225 (3)	
H8	0.8261	0.2737	0.3395	0.027*	
C9	0.68719 (12)	0.2674 (3)	0.40437 (9)	0.0200 (3)	
C10	0.68960 (12)	−0.0280 (3)	0.49192 (9)	0.0208 (3)	
C11	0.62985 (12)	0.4679 (3)	0.36912 (9)	0.0207 (3)	
C12	0.53195 (13)	0.5788 (3)	0.38160 (10)	0.0242 (4)	
H12	0.4803	0.5406	0.4196	0.029*	
C13	0.52194 (14)	0.7633 (3)	0.32661 (10)	0.0279 (4)	
H13	0.4620	0.8722	0.3206	0.033*	
C14	0.61369 (14)	0.7542 (3)	0.28479 (10)	0.0279 (4)	
H14	0.6291	0.8584	0.2440	0.034*	

### Atomic displacement parameters (Å^2^)

**Table:**

	*U*^11^	*U*^22^	*U*^33^	*U*^12^	*U*^13^	*U*^23^
F1	0.0259 (5)	0.0527 (7)	0.0494 (6)	0.0132 (5)	0.0156 (4)	−0.0045 (5)
N1	0.0169 (6)	0.0245 (7)	0.0224 (6)	0.0016 (5)	0.0036 (5)	0.0019 (5)
N2	0.0184 (6)	0.0232 (7)	0.0229 (6)	0.0012 (5)	0.0041 (5)	−0.0007 (5)
N3	0.0201 (7)	0.0296 (8)	0.0296 (7)	0.0035 (6)	0.0085 (5)	0.0081 (6)
O1	0.0253 (6)	0.0242 (6)	0.0270 (6)	0.0029 (5)	0.0073 (5)	0.0040 (5)
C1	0.0165 (7)	0.0256 (8)	0.0208 (7)	0.0008 (6)	0.0031 (6)	−0.0022 (6)
C2	0.0242 (8)	0.0277 (9)	0.0300 (8)	0.0039 (7)	0.0078 (6)	0.0032 (7)
C3	0.0280 (8)	0.0300 (9)	0.0330 (9)	0.0095 (7)	0.0063 (7)	0.0016 (7)
C4	0.0185 (7)	0.0379 (10)	0.0285 (8)	0.0071 (7)	0.0048 (6)	−0.0080 (7)
C5	0.0233 (8)	0.0341 (10)	0.0283 (8)	−0.0024 (7)	0.0100 (6)	−0.0026 (7)
C6	0.0230 (7)	0.0270 (8)	0.0272 (8)	0.0025 (7)	0.0052 (6)	0.0006 (7)
C7	0.0178 (7)	0.0225 (8)	0.0205 (7)	−0.0013 (6)	0.0020 (6)	−0.0018 (6)
C8	0.0201 (7)	0.0245 (8)	0.0236 (7)	0.0005 (6)	0.0052 (6)	0.0023 (6)
C9	0.0185 (7)	0.0210 (8)	0.0203 (7)	−0.0004 (6)	0.0013 (6)	−0.0023 (6)
C10	0.0179 (7)	0.0238 (8)	0.0206 (7)	−0.0004 (6)	0.0030 (6)	−0.0012 (6)
C11	0.0198 (7)	0.0218 (8)	0.0207 (7)	−0.0007 (6)	0.0033 (6)	−0.0015 (6)
C12	0.0211 (7)	0.0243 (8)	0.0272 (8)	0.0010 (6)	0.0036 (6)	−0.0025 (7)
C13	0.0271 (8)	0.0228 (8)	0.0324 (9)	0.0047 (7)	−0.0008 (7)	−0.0002 (7)
C14	0.0328 (8)	0.0216 (8)	0.0283 (8)	0.0035 (7)	0.0004 (7)	0.0051 (7)

### Geometric parameters (Å, °)

**Table:**

F1—C4	1.3623 (18)	C3—H3	0.9500
N1—C7	1.346 (2)	C4—C5	1.376 (3)
N1—C10	1.3503 (19)	C5—C6	1.391 (2)
N2—C10	1.346 (2)	C5—H5	0.9500
N2—C9	1.348 (2)	C6—H6	0.9500
N3—C10	1.359 (2)	C7—C8	1.387 (2)
N3—H31	0.902 (19)	C8—C9	1.390 (2)
N3—H32	0.87 (2)	C8—H8	0.9500
O1—C14	1.3667 (19)	C9—C11	1.456 (2)
O1—C11	1.3736 (18)	C11—C12	1.357 (2)
C1—C2	1.392 (2)	C12—C13	1.421 (2)
C1—C6	1.399 (2)	C12—H12	0.9500
C1—C7	1.491 (2)	C13—C14	1.344 (2)
C2—C3	1.388 (2)	C13—H13	0.9500
C2—H2	0.9500	C14—H14	0.9500
C3—C4	1.372 (2)		
			
C7—N1—C10	116.33 (13)	N1—C7—C8	121.46 (14)
C10—N2—C9	115.37 (12)	N1—C7—C1	116.36 (14)
C10—N3—H31	119.2 (12)	C8—C7—C1	122.18 (14)
C10—N3—H32	115.2 (12)	C7—C8—C9	117.68 (14)
H31—N3—H32	114.2 (17)	C7—C8—H8	121.2
C14—O1—C11	106.44 (12)	C9—C8—H8	121.2
C2—C1—C6	118.38 (14)	N2—C9—C8	122.34 (14)
C2—C1—C7	119.86 (14)	N2—C9—C11	116.84 (13)
C6—C1—C7	121.76 (14)	C8—C9—C11	120.82 (14)
C3—C2—C1	121.33 (16)	N2—C10—N1	126.82 (14)
C3—C2—H2	119.3	N2—C10—N3	117.09 (13)
C1—C2—H2	119.3	N1—C10—N3	116.08 (14)
C4—C3—C2	118.14 (16)	C12—C11—O1	109.78 (14)
C4—C3—H3	120.9	C12—C11—C9	133.89 (15)
C2—C3—H3	120.9	O1—C11—C9	116.32 (13)
F1—C4—C3	118.27 (16)	C11—C12—C13	106.53 (15)
F1—C4—C5	118.67 (15)	C11—C12—H12	126.7
C3—C4—C5	123.06 (15)	C13—C12—H12	126.7
C4—C5—C6	118.06 (15)	C14—C13—C12	106.75 (14)
C4—C5—H5	121.0	C14—C13—H13	126.6
C6—C5—H5	121.0	C12—C13—H13	126.6
C5—C6—C1	121.00 (16)	C13—C14—O1	110.49 (14)
C5—C6—H6	119.5	C13—C14—H14	124.8
C1—C6—H6	119.5	O1—C14—H14	124.8
			
C6—C1—C2—C3	2.2 (2)	C10—N2—C9—C11	179.48 (13)
C7—C1—C2—C3	−177.11 (14)	C7—C8—C9—N2	0.3 (2)
C1—C2—C3—C4	−1.0 (2)	C7—C8—C9—C11	−178.93 (13)
C2—C3—C4—F1	179.47 (14)	C9—N2—C10—N1	−0.3 (2)
C2—C3—C4—C5	−0.5 (2)	C9—N2—C10—N3	178.41 (13)
F1—C4—C5—C6	−179.16 (14)	C7—N1—C10—N2	−0.3 (2)
C3—C4—C5—C6	0.8 (2)	C7—N1—C10—N3	−178.93 (13)
C4—C5—C6—C1	0.4 (2)	C14—O1—C11—C12	0.17 (17)
C2—C1—C6—C5	−1.8 (2)	C14—O1—C11—C9	179.54 (13)
C7—C1—C6—C5	177.43 (14)	N2—C9—C11—C12	1.5 (2)
C10—N1—C7—C8	0.8 (2)	C8—C9—C11—C12	−179.20 (16)
C10—N1—C7—C1	−178.73 (12)	N2—C9—C11—O1	−177.67 (13)
C2—C1—C7—N1	10.0 (2)	C8—C9—C11—O1	1.6 (2)
C6—C1—C7—N1	−169.28 (13)	O1—C11—C12—C13	0.07 (17)
C2—C1—C7—C8	−169.55 (14)	C9—C11—C12—C13	−179.15 (16)
C6—C1—C7—C8	11.2 (2)	C11—C12—C13—C14	−0.29 (18)
N1—C7—C8—C9	−0.9 (2)	C12—C13—C14—O1	0.40 (18)
C1—C7—C8—C9	178.66 (13)	C11—O1—C14—C13	−0.36 (17)
C10—N2—C9—C8	0.2 (2)		

### Hydrogen-bond geometry (Å, °)

**Table:**

*D*—H···*A*	*D*—H	H···*A*	*D*···*A*	*D*—H···*A*
N3—H31···N2^i^	0.90 (2)	2.30 (2)	3.190 (2)	168 (2)
C5—H5···O1^ii^	0.95	2.58	3.474 (2)	157
C2—H2···N1	0.95	2.46	2.789 (2)	100

Symmetry codes: (i) −*x*+1, −*y*, −*z*+1; (ii) −*x*+2, *y*−1/2, −*z*+1/2.
